# Type D personality, mental distress, social support and health-related quality of life in coronary artery disease patients with heart failure: a longitudinal observational study

**DOI:** 10.1186/s12955-014-0204-2

**Published:** 2015-01-22

**Authors:** Margarita Staniute, Julija Brozaitiene, Julius Burkauskas, Nijole Kazukauskiene, Narseta Mickuviene, Robertas Bunevicius

**Affiliations:** Behavioral Medicine Institute, Lithuanian University of Health Sciences, Palanga, Lithuania

**Keywords:** Type D personality, Anxiety depression, Social support, Health-related quality of life, Coronary artery disease

## Abstract

**Background:**

The relationship between Type D personality and health related quality of life (HRQoL) in coronary artery disease patients is becoming more established, however, the factors that may explain this association remain unclear. The objective of the study was to examine the mediating effects of mental distress and social support on the relationship between the Type D personality and HRQoL in CAD patients with heart failure.

**Methods:**

A total of 855 CAD patients with heart failure were assessed on Type D personality, mental distress, perceived social support and HRQoL with the following self-administered questionnaires: the Type D personality scale - 14, the Hospital Anxiety and Depression scale, the Multidimensional Scale of Perceived Social Support and the Minnesota Living with Heart Failure Questionnaire.

**Results:**

The prevalence of Type D personality within the study population was 33.5%. Type D personality, anxiety symptoms, depressive symptoms and social support were all found to be determinants of decreased HRQoL (p’s < 0.001), once age, gender, NYHA functional class and acute myocardial infarction were adjusted for. Anxiety, depressive symptoms and social support were found to mediate the relationship between Type D personality and HRQoL. Type D personality exerted a stable effect on HRQoL over 24 months follow-up period.

**Conclusions:**

Type D personality has an independent significant effect on the HRQoL in CAD patients with heart failure, and this relation is mediated by anxiety and depressive symptoms, social support.

## Background

Coronary artery disease (CAD) remains a major public health problem [1] and is a major contributor to the progression of heart failure [2]. Approximately 1–2% of the adult population in developed countries suffers from heart failure, with prevalence rates rising to ≥10% among persons 70 years of age or older [3]. The overall prevalence of heart failure is increasing because of the ageing of the population, the success in prolonging survival in patients suffering coronary events, and the success in postponing acute coronary events by effective prevention in those at high risk [4]. Heart failure patients experience high levels of physical, functional and emotional distress, which has a major impact on the health-related quality of life (HRQoL) [5]. In an extensive research of both patients with CAD and patients with heart failure HRQoL was shown to be associated with disease severity [6,7], social support [8,9] and psychological factors, including symptoms of mental distress [10,11]. Moreover, more recent studies have reported that a Type D personality may present as chronic psychological risk factor among cardiac patients [12].

A distressed personality Type D profile is a vulnerability factor for general psychological distress that affects mental and physical health status [13]. Type D personality is characterized by a joint manifestation of negative affectivity and social inhibition and has been found to be an important determinant of outcomes in cardiac patients. Type D personality has a deleterious effect on the prognosis of patients with CAD. For example, Type D patients had a twofold increased risk of mortality and nonfatal myocardial infarction (MI) [14,15]. Type D personality is also a predictor of poor health status in patients with established CAD and heart failure. For example, type D personality has been linked to poor HRQoL in CAD patients undergoing cardiac rehabilitation [16], as well as in chronic heart failure patients [14]. In a prospective study, CAD patients with Type D personality were at a two-fold (OR = 2.2; 95% CI: 1.2–3.8) increased risk for reporting poor perceived health at 5-year follow-up when compared with non-Type D CAD patients [17].

Type D personality trait is associated with numerous adverse behavioral and biological traits that can predispose towards progression of heart failure and worse patient outcomes [18]. For example, it has been demonstrated in patients with established heart failure, that Type D personality is associated increased risk for poor treatment adherence [19] and greater serum inflammatory marker concentrations [20].

Type D personality is defined as having a high score on two stable personality traits, negative affectivity and social inhibition [21,22]. Inhibited individuals are more vulnerable for developing anxiety and therefore Type D personality patients are at risk to experience increased levels of anxiety symptoms [23]. In heart failure patients, Type D personality, but not depressive symptoms predicted clinically significant anxiety at 1-year follow-up. These findings suggest that assessment of type D status could be used to identify heart failure patients at high risk for future anxiety that can subsequently contribute towards impaired quality of life [23].

Type D personality patients experience increased levels of depressive symptoms [24,25]. For example, in one-year follow-up study of heart failure patients, Type D personality trait was independently associated with greater depressive symptom severity [26]. It is well established that depression is associated with adverse cardiac outcomes such as increased mortality [27], decreased quality of life [10,11] and is an independent risk factor in CAD [28].

Patients with Type D personality have a tendency to not share emotions in social interactions, due to a fear of rejection or disapproval, and have a perceived lack of social support. It was shown that CAD patients with Type D personality, compared with non-Type D individuals, reported less perceived social support. The influence of Type D personality was apparent even controlling these finding for anxiety and depression [29].

Social support is a known buffer of psychological distress and has also been shown to influence adverse medical outcomes. Lack of social support is associated with increased morbidity and mortality in patients with CAD and heart failure [30-32] and with lower levels of HRQoL, especially in women [9].

Although mental distress and lack of social support are strongly related to Type D personality, it remains unclear if these factors may mediate the association between Type D personality and HRQoL. Type D personality is not a pathological condition in itself, rather it is a normal personality disposition that is stable over a long period of time [33]. Therefore, more research is necessary to determine which factors may mediate the adverse effects of Type D personality upon outcomes in cardiac patients.

From a clinical perspective, it is important to know if heart failure patients with Type D personality trait are at greater risk for continuous deterioration/impairment in their HRQoL, as this knowledge could potentially allow for more accurate risk-stratification of heart failure patients admitted to rehabilitation program and provide with an opportunity for early interventions aiming to address behavioral/psychological risk factors in order to improve patient prognosis.

The aim of this study was to evaluate the impact of Type D personality upon HRQoL in CAD patients with heart failure after acute coronary syndromes, both during cardiac rehabilitation and during the two years follow-up. Moreover, this study aimed, to examine the role of mental distress and social support as psychosocial factors that may mediate the association between Type D personality and HRQoL.

## Methods

### Sample population

In a period from 2010 to 2013 a total of 962 consecutive CAD patients attending an in-patient cardiac rehabilitation program at the Cardiovascular Rehabilitation Clinic of the Behavioral Medicine Institute of the Lithuanian University of Health Sciences in Palanga, Lithuania were invited to participate in this study. All study patients were admitted to the rehabilitation program within one week after treatment for acute coronary syndromes. Eligible patients were those who had a clinical diagnosis of heart failure, according to European Society of Cardiology guidelines [34,35]. Study cardiologist (J.B.) evaluated patients’ symptoms of heart failure and objective evidence of cardiac dysfunction assessed by echocardiography and invited patients to participate. After agreeing to participate, written informed consent (WIC) was given for evaluating socio-demographic and clinical data and for using personal data (name, address and phone numbers) for follow up assessment. Patients were excluded from the present study if they had cognitive disorientation or communicative disabilities (n = 51), had severe comorbidities (n = 18) or unstable cardiovascular status (n = 28), were not willing to participate in the study (n = 10). Following the exclusion criteria the study population comprised 855 (74% men and 26% women; mean age, 58 ± 9 years) patients with CAD. As we longitudinally observed patients’ HRQoL records, we had 30% (265 patients) overall attrition leaving 650 patients after 6 months, 651 patients after 12 months, 648 patients after 18 months and 590 patients after 24 months since the study started. The study protocol was approved by The Regional Medical Research Ethics Committee.

### Study design

**Figure 1:**
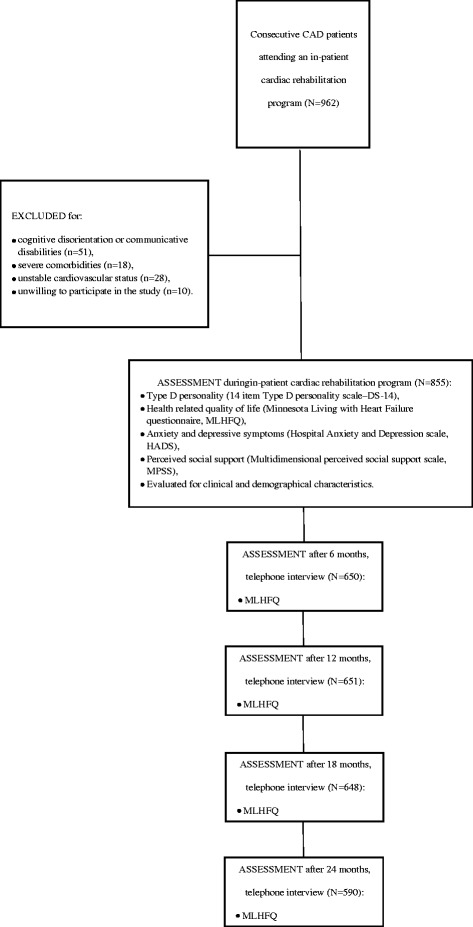
**Participant flow chart.**

The baseline measure was chosen to be patients’ assessment within three days of admission to the rehabilitation program after they signed WIC. During that time study cardiologist evaluated patients demographic and clinical characteristics. During the same time, patients without presence of medical professionals in separate room in the clinic filled-in questionnaires for assessment of Type D personality [36], HRQoL [37], anxiety and depressive symptoms [38] and perceived social support [39]. These were validated Lithuanian versions of self-rating scales [9,22,40]. HRQoL [37] was evaluated by qualified nurse at baseline and after 6-, 12-, 18-, and 24-months follow-up using telephone interview (Figure 1).

### Measures

#### Socio-demographic and clinical characteristics

Age, sex and marital status (living alone, living with a partner) were reported by patients during the initial interview with study cardiologist.

Study cardiologist evaluated symptoms of heart failure (at rest and during exercise test), objective evidence of cardiac dysfunction (left ventricular systolic and/or diastolic) and heart failure characteristics. The NYHA functional classification system was used by the cardiologist to classify the degree of functional disability based on symptoms of fatigue, palpitation or dyspnea and activity limitations [41]. NYHA Class I referring to no limitation, Class II to slight limitation, Class III to marked limitation in patient’s physical activity respectively. Class IV represents patients who are unable to carry out any physical activity without discomfort.

Left ventricular ejection fraction (LVEF) was assessed by echocardiography and classified into: 1) LVEF ≤ 40% - moderate to severe systolic dysfunction, 2) LVEF > 40% - normal to mild systolic dysfunction. Patient’s history of MI, acute MI, angina pectoris and hypertension were retrieved from the medical records.

### Type D personality scale

Type D personality was measured with the Type D Personality Scale (DS14) validated in Lithuania [22]. The DS14 consists of two 7-item subscales assessing negative affectivity (NA) and social inhibition (SI). Items on both 7-item scales are answered on a 5-point Likert scale from “false” (0) to “true” (4) with scores ranging from 0 to 28 on each subscale. Scores equal or greater than 10 on both DS14 subscales of NA and SI indicate Type D personality. Reliability of the scale is acceptable with Cronbach’s alpha of 0.84/0.75 for the NA and SI subscales.

### Health-related quality of life scale

HRQoL was evaluated using the Minnesota Living with Heart Failure Questionnaire (MLHFQ). MLHFQ is a commonly used measure of HRQoL, specifically designed for persons with heart failure. This questionnaire consists of 21 questions covering physical, socioeconomic and psychological dimensions of life, relative to the limitations frequently associated with the profile of cardiac insufficiency [37]. Items are answered on a 6-point Likert scale from 0 (no) to 5 (very much). The final score is the sum of the responses and varies from 0 (no impairment) to 105 (total impairment). The higher scores reflect a decreased HRQoL. MLHFQ has adequate internal reliability, Cronbach’s alpha coefficients was 0.81.

### Anxiety and depression symptoms scale

Anxiety and depressive symptoms were assessed using the HADS. The HADS is a 14-item self-rating instrument that consists of subscales of anxiety (HADS-A) and depression (HADS-D), which are designed to measure respective symptoms. Each item is rated on a scale from 0 to 3 based on the subject’s experience over the past week. Total scores on the HADS-A and HADS-D range from 0 to 21, with higher scores indicating more severe anxiety and depression symptoms. Patients were considered to have anxiety or depressive symptoms if they scored ≥ 8 on the HADS-D or HADS-A [42]. The Lithuanian version of the HADS has been shown to be reliable screening instrument in Lithuanian CAD patients. In the current study, Cronbach’s coefficients alpha of the HADS-A subscale was 0.84 and 0.75 for HADS-D subscale, suggesting adequate internal consistency.

### Perceived social support scale

Perceived social support was assessed using Multidimensional Scale of Perceived Social Support (MSPSS). The MSPSS assesses the perceived adequacy of social support from family, friends and significant others as rated by the patient [39]. The MSPSS is comprised of 12 items of social support scored on a seven-point Likert-type scale ranging from “very strongly disagree” to “very strongly agree”. For the purposes of this study, only total score was calculated. For the total score, ratings are summed and divided by 12. Total scores range from 1 to 7, with higher scores indicating a higher perception of available social support. The MSPSS had adequate internal reliability, with an alpha coefficient of 0.88.

### Statistical analysis

Differences in clinical, demographic, mental distress and HRQoL characteristics of patients, stratified by Type D personality were tested using independent sample t-tests, Fischer Exact test and in cases where *p* was lower than 0.05 difference between proportions test.

Simple univariate regression analyses were conducted to determine the relationship between HRQoL and gender, age, acute MI, angina pectoris, LVEF, NYHA class, hypertension, Type D, anxiety score, depression score, and social support. A probability level of 0.05 or less was used as the criterion to include the independent variable in the multivariate models and a probability level of 0.1 or more was used as the criterion to remove the independent variable from the models. Thus, we created three models entering statistically significant socio-demographic characteristics as independent variables in the first model, adding significant clinical characteristics in the second model and significant mental distress characteristics as well as perceived social support in the third model.

The residual scatterplots were examined to check the assumptions of normality, linearity and homoscedasticity between the predicted dependent variable scores and errors of prediction, and the assumptions were deemed to be satisfied. Furthermore, the Durbin-Watson test statistic expressed no correlation in adjacent residuals and the variance inflation factor (VIF) and tolerance statistic indicated no problem with multicollinearity.

The repeated measures analysis of variance (ANOVA) was used to assess effect of Type D personality and follow-up time on HRQoL with adjustment for potential confounders, such as age, gender, NYHA class and acute MI. For this task statistical analysis was performed only patients participating in all monitoring phases over a 24 month period.

The changes in HRQoL during follow-up were assessed by effect size, which is defined as the difference between two means divided by a standard deviation for the data. An effect size of 0.2 is considered a “small” effect; 0.5, a “moderate” effect; and 0.8 to infinity, a “large” effect. Differences in HRQoL between Type D and non-Type D personality patients at the beginning of cardiac rehabilitation program (T1), after 6- (T6), 12- (T12), 18- (T18) and 24- (T24) months and effect size, using *T*-test for independent samples was estimated. Differences in HRQoL within Type D and non-Type D patients groups over time between T1 and T6, T6 and T12 and T6 and T18, T6 and T24, using *T*-test for dependent samples and estimation magnitude of change with an effect size was performed.

Mediation analysis was used to examine whether mental distress or perceived social support mediated the link between Type D personality and HRQoL after controlling for covariates (gender, age, NYHA, acute MI) [43]. Analysis was performed following conditions that must be fulfilled in order to establish mediation effect: first, the predictor variable (Type D personality) must significantly influence the mediator variable (anxiety symptoms, depressive symptoms or social support); second, the predictor variable must significantly influence the outcome variable HRQoL and third, the predictor variable’s influence on the outcome variable must be reduced or become non-significant when the mediator variable is included in the model. To test significance of the indirect path via the mediator variable, we used Sobel’s test applying a utility provided by Preacher and Leonardelli [44].

Data was analyzed with the SPSS 17.0 for Windows (Chicago, IL). Data are presented as mean ± standard deviation for continuous variables and as the number (percent) for categorical variables.

## Results

### Baseline characteristics

There were no differences between participants who were interviewed vs. those who were not interviewed (i.e., those whom we were unable to contact, who had refused, or who were excluded at the interview) on basic clinical and demographic characteristics. We conducted a series of *t* test comparisons using the clinical information obtained during the screen of the electronic medical record on behalf of providers. We found no significant differences in age, number of comorbid conditions and minimum LVEF (of those values in the clinical record) between those interviewed and those who were not interviewed (all p values > 0.05).

In short, the majority of studied patients (80%) had hypertension: grade 1 (mild) 6.4%, grade 2 (moderate) 64.2%, grade 3 (severe) – 9.4%. Twenty eight percent had angina pectoris, 59% – experienced an acute MI, 13% – had a past history of MI. Six percent of patients were classified NYHA functional class I, 77% – NYHA class II and 17% – NYHA class III, there were no patients classified as NYHA IV class in this study. Majority of study patients 688 (86%) had normal to mild systolic dysfunction (LVEF > 40%) and 112 (14%) had moderate to severe systolic dysfunction (LVEF ≤ 40%). The prevalence of patients with moderate-severe depressive (HADS-D score ≥ 8) and anxiety (HADS-A score ≥ 8) symptoms was 13% and 33%, respectively, within the study population.

The prevalence of patients with Type D personality was 33.5%. Clinical, demographic, mental distress and HRQoL characteristics of patients, stratified by Type D personality, are presented in Table 1. Type D personality patients were more likely to be female, have a greater NYHA class, have more anxiety and depressive symptoms, have a lack of social support and have decreased HRQoL compared to non-Type D patients.

**Table 1:** **Characteristics of all patients at inclusion stratified by Type D personality**

	**All patients**	**Type D**	**Non Type D**	**P value**	**Difference between proportions, %**
	**(n = 855)**	**(n = 286)**	**(n = 569)**		
Age, years (mean ± SD)	58.6 ± 8.8	58.6 ± 8.8	57.9 ± 9.0	0.304	
Gender, n (%)				<0.001	
Men	633 (74.0)	182 (63.6)	451 (79.3)	<0.001	15.6%
Women	222 (26.0)	104 (36.4)	118 (20.7)	0.010	
Marital status, n (%)				0.336	
Living with a partner	697 (81.5)	228 (79.7)	469 (82.4)	0.389	2.7%
Living alone	158 (18.5)	58 (20.3)	100 (17.6)	0.675	
Diagnosis, n (%)				0.669	
Angina pectoris	240 (28.1)	83 (28.9)	157 (27.5)	0.667	1.4%
Acute myocardial infarction	506 (59.2)	169 (59.1)	337 (59.2)	0.978	0.1%
Previous myocardial infarction	109 (12.7)	34 (11.9)	75 (13.2)	0.591	1.3%
Hypertension, n (%)				0.109	
Grade 1 (mild)	55 (6.4)	18 (6.3)	37 (6.5)	0.911	0.2%
Grade 2 (moderate)	549 (64.2)	194 (67.8)	355 (62.4)	0.121	5.4%
Grade 3 (severe)	80 (9.4)	23 (8.0)	57 (10.0)	0.343	0.2%
NYHA class, n (%)				0.027	
I	52 (6.1)	17 (5.9)	35 (6.2)	0.863	0.2%
II	659 (77.1)	207 (72.4)	452 (79.4)	0.022	7.1%
III	144 (16.8)	62 (21.7)	82 (14.4)	0.007	7.3%
Mental distress					
HADS-A ≥ 8, n(%)	281 (32.9)	164 (57.3)	117 (20.6)	<.001	36.8%
HADS-D ≥ 8, n(%)	112 (13.1)	82 (28.7)	30 (5.3)	<.001	23.4%
Perceived social support, mean score ± SD	6.1 ± 1.0	5.9 ± 1.1	6.2 ± 0.9	<.001	
Minnesota Living with heart failure questionnaire, mean score ± SD	31.7 ± 20.0	40.3 ± 19.6	27.3 ± 18.8	<.001	

NYHA – New York Heart Association; HADS-D - Hospital Anxiety and Depression scale depression subscale; HADS-A - Hospital Anxiety and Depression scale anxiety subscale.

### Associations among Type D personality, clinical characteristics, mental distress, social support, and HRQoL

Univariate linear regression analyses showed that Type D personality was associated with more anxiety symptoms (β = 0.41, p < 0.001), more depressive symptoms (β = 0.42, p < 0.001) and low perceived social support (β = −0.162, p < 0.001) (data not shown).

However, presence of Type D personality, and greater anxiety and depressive symptom severity were significantly associated with worse HRQoL, following adjustment for socio-demographic and clinical characteristics (Table 2). In addition, older age, female gender, more severe NYHA class, and acute MI were each associated with worse HRQoL.

**Table 2:** **Mental distress and social support as determinants of health-related quality of life**

		**Health related quality of life**		**Depressive symptoms**		**Anxiety symptoms**		**Social support**	
		**β**		**β**		**β**		**β**	
**Model 1**	R^2^		0.07***		0.07***		0.09***		0.02**
	ΔR^2^		0.07***		0.07***		0.09***		0.02**
Age		0.08		0.15***		-0.03		-0.07*	
Gender		0.26***		0.20***		0.31***		-0.09*	
**Model 2**	R^2^		0.11***		0.08***		0.11***		0.02**
	ΔR^2^		0.04***		0.01*		0.02**		0.001
Age		-0.11**		0.12***		-0.06		-0.08*	
Gender		0.24***		0.18***		0.29***		-0.09*	
Acute MI		-0.15***		-0.02		-0.07		0.00	
NYHA		0.17***		0.10**		0.12**		0.04	
**Model 3**	R^2^		0.30***		0.46***		0.43***		0.09***
	ΔR^2^		0.19***		0.38***		0.32***		0.07***
Age		-0.13***		0.14***		-0.12***		-0.04	
Gender		0.12***		-0.01		0.18***		-0.04	
Acute myocardial infarction		-0.13***		0.01		-0.05*		-0.01	
NYHA		0.11***		0.03		0.06*		0.06	
Anxiety symptoms		0.20***		0.48***		–		0.02	
Depressive symptoms		0.25***		–		0.50***		-0.27***	
Social support		-0.04		-0.16***		0.01		–	
Type D personality		0.09*		0.21***		0.16***		-0.03	

*p value < 0.05; **p value < 0.01; ***p value < 0.001; NYHA – New York Heart Association functional class.

### HRQoL changes two years follow-up

**Figure 2:**
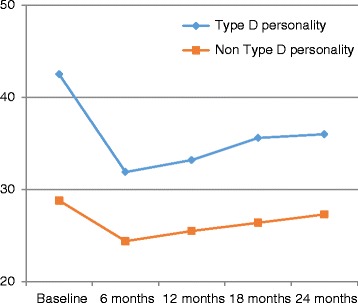
**Minnesota Living with heart failure global score stratified by Type D personality over two years.**

The dynamic of quality of live over 2-years period in Type D personality patients and non-Type D personality patients is showed in Figure 2. During the period from baseline to 6 month follow greater improvement of HRQoL was documented in Type D than non-Type D patients (effect sizes from T1 to T6 at levels of 0.63 and 0.24, respectively) (Table 3). The same pattern was observed comparing Type D and non-Type D patients’ results in HRQoL in all phases of the two year follow-up (from T6 to T18 effect sizes of -0.12 and -0.07 respectively; from T6 to T18 effect sizes of -0.24 and -0.14 respectively; and from T6 to T24 effect sizes of -0.30 vs -0.21, respectively). However, in Type D personality patients HRQoL remained worse over all follow-up periods when comparing with non-Type D patients (Tables 3 and 4).

**Table 3:** **Differences of HRQoL and effect sizes (ES) between separate follow-up periods**

**Health related quality of life**		**Type D personality**			**Non-Type D personality**		
		**Mean ± SD**	**p**	**ES(d)**	**Mean ± SD**	**p**	**ES(d)**
**Periods**							
T1	Baseline	42.45 ± 19.78	<0.001	.63	28.84 ± 19.08	<0.001	0.24
T6	6 month	31.96 ± 16.52			24.40 ± 14.13		
T6	6 months	31.96 ± 16.52	0.255	-0.12	24.40 ± 14.13	0.162	-0.07
T12	12 month	33.27 ± 17.59			25.49 ± 15.04		
T6	6 months	31.96 ± 16.52	0.006	-0.24	24.40 ± 14.13	0.010	-0.14
T18	18 month	35.68 ± 17.54			26.45 ± 14.66		
T6	6 months	31.96 ± 16.52	0.002	-0.30	24.40 ± 14.13	0.001	-0.21
T24	24 month	36.05 ± 17.10			27.33 ± 14.73

**Table 4:** **Differences of HRQoL and effect sizes during follow-up periods**

**Health related quality of life**	**Type D personality**	**Non-type D personality**	**P**	**ES(d)**
	**Mean ± SD**	**Mean ± SD**		
Baseline	42.45 ± 19.78	28.84 ± 19.08	<0.001	0.74
6 months	31.96 ± 16.52	24.40 ± 14.13	<0.001	0.47
12 months	33.27 ± 17.59	25.49 ± 15.04	<.001	0.50
18 months	35.68 ± 17.54	26.45 ± 14.66	<0.001	0.58
24 months	36.05 ± 17.10	27.33 ± 14.73	<0.001	0.58

The repeated measures analysis of variance showed, that Type D personality was a significant independent determinant of HRQoL F(1.426) = 4.00; p = 0.046), adjusting for gender, age and NYHA class, acute MI. In the adjusted analysis, the within-subjects effect for time was not significant F(1.426) = 4.00; p = 0.463), indicating Type D and Non-Type D personality patient groups do not change in HRQoL over time Thus, Type D personality exerted a stable effect on HRQoL over time.

### Mental distress and social support as mediators

**Figure 3:**
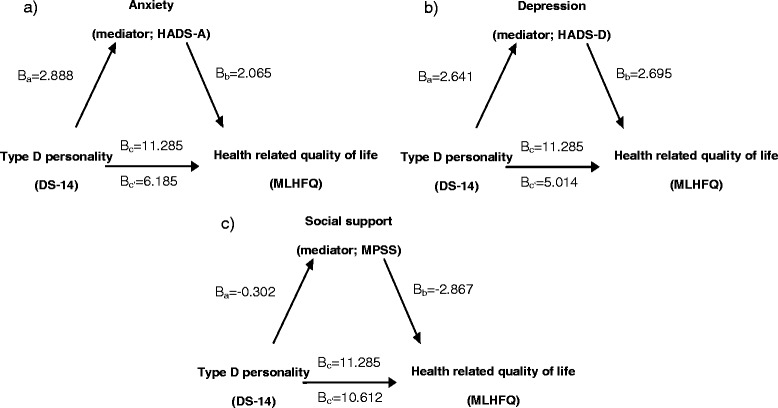
**Relation between Type D personality, possible mediators and health related quality of life.** **a)** Mediation model relating Type D personality, anxiety, and health related quality of life. **b)** Mediation model relating Type D personality, depression, and health related quality of life. **c)** Mediation model relating Type D personality, social support, and health related quality of life.

The predictor variable (Type D personality) significantly influenced the proposed mediators (anxiety, depressive symptoms and social support) in a linear regression model. For anxiety symptoms the unstandardized regression coefficient B_a_ was 2.888 (p < 0.001, standard error SE = 0.242); for depressive symptoms B_a_ = 2.641 (p < 0.001, SE = 0.198); for social support B_a_ = −0.302 (p < 0.001, SE = 0.076). The coefficients used for testing mediation are also shown in Figure 3.

The predictor variable Type D personality significantly influenced the outcome variable HRQoL (step 1 in Table 5); the unstandardized coefficient B_c_ was 11.285 (p < 0.001, SE = 1.349). When adding the possible mediator anxiety symptoms to the linear regression model (step 2 in Table 5), the HRQoL coefficient decreased significantly to B_c’_ = 6.185 (SE = 1.382) suggesting mediation effect. Anxiety symptoms significantly influenced HRQoL with a coefficient B_b_ of 2.065 (p < 0.001, SE = 0.169). The indirect pathway was significant at p < 0.001, based on Sobel’s test.

**Table 5:** **Mental distress and social support as mediators of the relationship between Type D personality and health-related quality of life**

	**Health related quality of life (outcome)**					
	**Step 1**			**Step 2**		
				**Type D personality (predictor) x mediator**		
	**B [95%-CI]**	**β**	**R** ^**2**^	**B [95%-CI]**	**β**	**R** ^**2**^
Type D personality (predictor)	11.285[8.638;13.933]	0.267***	0.177			
Anxiety symptoms (mediator)	2.065[1.733;2.397]	0.389***	0.243	1.758[1.403;2.113]	0.331***	0.261
Depressive symptoms (mediator)	2.695[2.302;3.089]	0.415***	0.267	2.365[1.935;2.796]	0.036***	0.278
Social support (mediator)	2.867[−4.107;−1.627]	−0.148***	0.129	−2.192[−3.401;−0.982]	−0.113***	0.189

***p value < .001.

The same analysis was done using depressive symptoms as mediator variable. Again, the predictor variable significantly influenced HRQoL (step 1 in Table 5). After adding depressive symptoms to the model, the unstandardized coefficient decreased to B_c’_ = 5.014 (p < 0.001, SE = 1.391) (step 2 in Table 5). Depressive symptoms still exerted significant influence on the HRQoL with a coefficient B_b_ = 2.695 (p < 0.001, SE = 0.201). The indirect path was significant at p < 0.001, according to Sobel’s test.

The last analysis included social support as mediator variable. As in previous models Type D personality significantly influenced HRQoL (step 1 in Table 3). Adding social support to the model (step 2 in Table 5), the HRQoL coefficient decreased significantly to B_c’_ = 10.612 (SE = 1.353) suggesting mediation effect. Social support significantly influenced HRQoL B_b_ = −2.867 (p < 0.001, SE = 0.632). The indirect path was significant at p < 0.001, according to Sobel’s test.

Assessment of overall strength of our regression model showed that the addition of the mediator variables anxiety symptoms, depressive symptoms and social support to the independently generated model greatly increased R^2^: when using anxiety symptoms as mediator, R^2^ increased from 0.243 to 0.261; for depressive symptoms, R^2^ increased from 0.267 to 0.278; and when using social support, R^2^ increased from 0.129 to 0.189 (Table 5).

## Discussion

In CAD patients with heart failure undergoing rehabilitation and during a two year follow-up, Type D personality was significantly associated with impaired HRQoL, independent from age, gender and disease severity. In addition, the present results may provide information regarding the mechanisms underlying this association. Specifically, our findings indicate that mental distress and perceived social support may partially account for the relation between Type D personality and HRQoL. Our findings showed that one third of CAD patients with heart failure were classified as having a Type D personality. Given the fact that these individuals have a significantly impaired quality of life, it is very important to establish modifiable factors that may have impact on Type D personality.

Previous studies exploring the association of Type D personality with HRQoL in cardiac patients also have shown that Type D personality is associated with impaired HRQoL, after adjusting for potential confounders related to HRQoL, including depressive symptoms [21,45-47].

One possible explanation for why Type D personality patients had a decreased HRQoL may be that Type D personality patients are at higher risk for poor medication adherence, which may lead to adverse health outcomes [48]. Type D personality carries an increased risk of mental disorders, such as depression [21], and symptoms of anxiety [23], thereby, increasing the emotional burden of CAD.

In this study Type D personality was consistently associated with impaired HRQoL over the period of 24 months. In previous studies [16,21,45] similar findings were found, however the follow up periods were shorter in duration: from 9 to 12 months. Thus, the current study extends the findings of these studies by showing that Type D personality had a stable effect over a longer 24-months period.

To the extent of our knowledge, there are no studies that examined Type D personality mediating factors in CAD patients with heart failure. One study examined HRQoL in coronary artery bypass graft surgery patients 6 months after operation and found that increased levels of anxiety largely mediated the influence of Type D personality on no change deterioration in HRQoL, whereas increased symptoms of depression explained deterioration in HRQoL without the influence of Type D personality [45]. A cross-sectional study of tinnitus patients found that Type D personality was also a direct predictor of decreased HRQoL and this influence was mainly mediated by symptoms of depression and anxiety [47]. The present study, showed similar results that Type D personality remains significant determinant of impaired HRQoL with both mediating mental distress factors.

Social support is a known buffer of psychological distress, our study results showed that perceived social support also mediates the relationship between Type D personality and HRQoL. It is important to note that support perceptions (perceived support) and receipt of supportive behaviors (received support) are different characteristics. According to meta-analytical review only perceived support, but not received support, has been linked to health-related outcomes [49]. What is more patients with lower perceived social support tend to be more depressed [50] and anxious [51]. Symptoms of depression and anxiety may inhibit Type D personality characteristics leading to decrease in HRQoL later on [52]. On the other way round anxiety and depression in Type D personality patients make them avoid expressing appropriate and timely emotions. Avoiding express emotions can reduce the quality of interpersonal relationships and HRQoL. Our findings add to the knowledge of perceived social support being mediator between Type D personality and HRQoL. Whether this particular effect is the same in other types of support, such as received social support, is unknown.

However our results with regards to mediation effect should be interpreted with caution. Type D personality influence on HRQoL was significant, but only slightly reduced when anxiety symptoms and social support (but not depression symptoms) were included in the models. This often suggests evidence of the effects of one or more omitted mediators. It could be also interpreted as a sign of some as-yet-undiscovered mediation mechanism [53] e.g. specific illness perceptions [54].

As to test this hypothesis was beyond the scope of our aims, future studies regarding mechanisms responsible for the relationship between Type D personality and HRQoL are warranted.

Type D personality patients deserve special attention during the period of rehabilitation as it was shown that Type D personality independently predicts a decreased HRQoL. Rehabilitation offers an excellent opportunity for the identification of mental distress and the opportunities for psychological interventions, such as depression and anxiety management. Expanded cardiac rehabilitation reduces Type D score, anxiety and depressive symptoms and improves the quality of life [55].

The findings of this study should be interpreted with some caution, in terms of generalizability, as the majority of study CAD patients had mild to moderate heart failure and all study patients attended cardiac rehabilitation program. Thus, the results may not apply to patients with more advanced heart failure and those who do not attend cardiac rehabilitation programs. Furthermore, lack of more comprehensive assessment of mental health status precluded from more rigorous assessment of mental status dynamics of CAD patients. On the other hand, the major strengths of our study include a large sample size, longitudinal observational study design and the use of reliable and valid measures.

## Conclusions

The present study demonstrated that Type D personality CAD patients with heart failure reported impaired HRQoL, during phase II cardiac rehabilitation and two years follow-up. Depressive, anxiety symptoms and perceived social support may mediate this association. For this reason, when planning cardiac rehabilitation programs vulnerable patients, such as those with Type D personality should be targeted for psychological intervention with the aims of improving HRQoL.

## Abbreviation

CAD: Coronary artery disease
HRQoL: Health-related quality of life
NYHA: New York Heart Association
DS14: Type D personality scale
NA: Negative affectivity
SI: Social inhibition
MLWHFQ: Minnesota Living With Heart Failure Questionnaire
HADS: Hospital Anxiety and Depression scale
HADS-A: Hospital Anxiety and Depression scale anxiety subscale
HADS-D: Hospital Anxiety and Depression scale depression subscale
MSPSS: Multidimensional Scale of Perceived Social Support
